# Repurposing Terfenadine as a Novel Antigiardial Compound

**DOI:** 10.3390/ph16091332

**Published:** 2023-09-21

**Authors:** Daniel Osmar Suárez-Rico, Francisco Javier Munguía-Huizar, Rafael Cortés-Zárate, José Manuel Hernández-Hernández, Sirenia González-Pozos, Armando Perez-Rangel, Araceli Castillo-Romero

**Affiliations:** 1 Departamento de Fisiología, Centro Universitario de Ciencias de la Salud, Universidad de Guadalajara, Calle Sierra Mojada 950, Independencia Oriente, Guadalajara 44340, Mexico; dosuarezr94@gmail.com; 2 Departamento de Microbiología y Patología, Centro Universitario de Ciencias de la Salud, Universidad de Guadalajara, Sierra Mojada 950, Col. Independencia, Guadalajara 44340, Mexico; paco112009@hotmail.com (F.J.M.-H.); rcortesz@hotmail.com (R.C.-Z.); 3 Departamento de Biología Celular, Centro de Investigación y Estudios Avanzados del Instituto Politécnico Nacional, Av. Instituto Politécnico Nacional 2508, Col. San Pedro Zacatenco, Ciudad de Mexico 07360, Mexico; manuel.hernandez@cinvestav.mx (J.M.H.-H.); rangelarm@yahoo.com (A.P.-R.); 4 Unidad de Microscopía Electrónica LaNSE, Centro de Investigación y Estudios Avanzados del Instituto Politécnico Nacional, Av. Instituto Politécnico Nacional 2508, Col. San Pedro Zacatenco, Ciudad de Mexico 07360, Mexico; sgonzale@cinvestav.mx

**Keywords:** *Giardia lamblia*, terfenadine, ion channels, tubulin, drug repurposing, autophagy

## Abstract

*Giardia lamblia* is a highly infectious protozoan that causes giardiasis, a gastrointestinal disease with short-term and long-lasting symptoms. The currently available drugs for giardiasis treatment have limitations such as side effects and drug resistance, requiring the search for new antigiardial compounds. Drug repurposing has emerged as a promising strategy to expedite the drug development process. In this study, we evaluated the cytotoxic effect of terfenadine on *Giardia lamblia* trophozoites. Our results showed that terfenadine inhibited the growth and cell viability of *Giardia* trophozoites in a time–dose-dependent manner. In addition, using scanning electron microscopy, we identified morphological damage; interestingly, an increased number of protrusions on membranes and tubulin dysregulation with concomitant dysregulation of *Giardia GiK* were observed. Importantly, terfenadine showed low toxicity for Caco-2 cells, a human intestinal cell line. These findings highlight the potential of terfenadine as a repurposed drug for the treatment of giardiasis and warrant further investigation to elucidate its precise mechanism of action and evaluate its efficacy in future research.

## 1. Introduction

*Giardia lamblia*, a flagellated protozoan with ubiquitous distribution, is the causative agent of giardiasis. This parasitosis is highly infectious and difficult to eradicate. Most of the cases are asymptomatic, but symptomatic infections typically include stomach cramps, bloating, nausea, and greasy diarrhea [1,2]. Most people recover fully without any long-lasting effects, but a small percentage may experience persistent or recurring symptoms, such as the following: postinfection irritable bowel syndrome, malabsorption and nutritional deficiencies, poor cognitive function, and reactive arthritis associated with an exaggerated immunologic response [3,4]. The current treatment of giardiasis is based on 5-nitroimidazole derivatives and some benzimidazoles [5,6]. However, they cause adverse effects such as allergic reactions and severe harm including carcinogenic activity, neurotoxic effects, and hepatic failure [7,8,9,10]. The side effects, adverse drug reactions, emergence of drug resistance, and increase in therapeutic failures have stimulated an increasing demand for novel antigiardial compounds [11,12,13].

**Figure 1:**
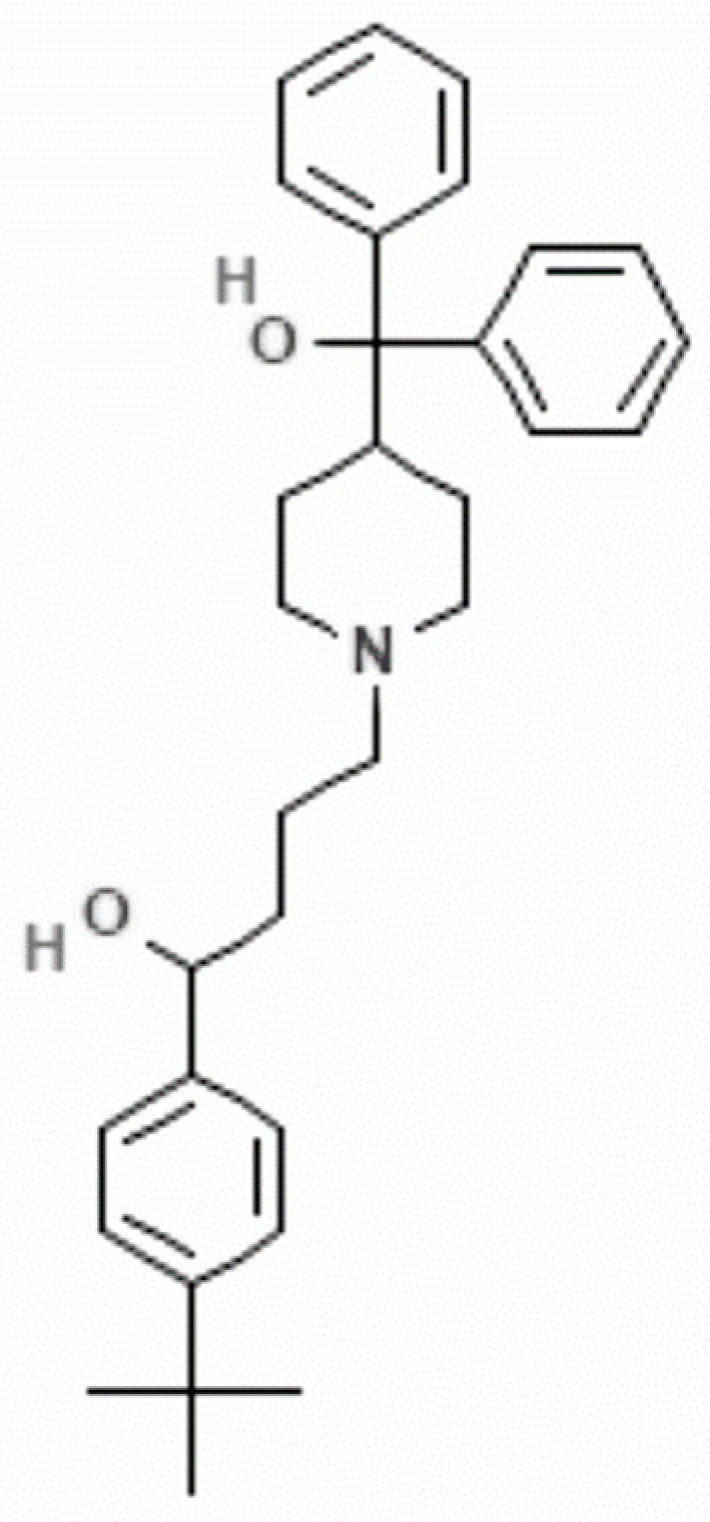
Chemical structure of terfenadine (α-(4-tert-butylphenyl)-4-hydroxydiphenylmethyl)-1-piperidinebutanol) [20].

Currently, drug repurposing is a key approach in drug development, and the use of medications (approved by the FDA) with known safety profiles offers results in less time. Some examples of recent findings suggest that disulfiram, which is a drug originally developed to treat chronic alcoholism, and acetylsalicylic acid, which is widely used to reduce pain, fever, and inflammation, are highly active against *Giardia* trophozoites [14,15], but they are still not in clinical use. Auranofin, a drug used for rheumatoid arthritis treatment, became interesting to use for a wider variety of diseases when it was discovered to be highly effective against cancer and infectious diseases including severe acute respiratory syndrome SARS-CoV-2. In addition, as an antiparasitic drug, this gold compound has been shown to be a good candidate treatment for amebiasis and giardiasis, but the mechanism of action of this drug is still not completely understood and has many potential gastrointestinal side effects [16,17]. Previously, we showed terfenadine (Figure 1), an antihistamine drug from the piperidine family that is used for the treatment of allergic conditions, to be a promising compound against *amebiasis* as it affects the growth and pathogenicity of *Entamoeba histolytica* [18]. In *G. lamblia*, homology modeling and molecular docking analysis predicted 13 binding sites for terfenadine in the pore-blocking site of a putative potassium ion channel called *GiK* (GL50803_101194) [19]. In this study, we describe the cytotoxic effect of terfenadine on *Giardia* trophozoites.

## 2. Results

### 2.1. Terfenadine Inhibits Growth and Cell Viability of *Giardia lamblia* Trophozoites

**Figure 2:**
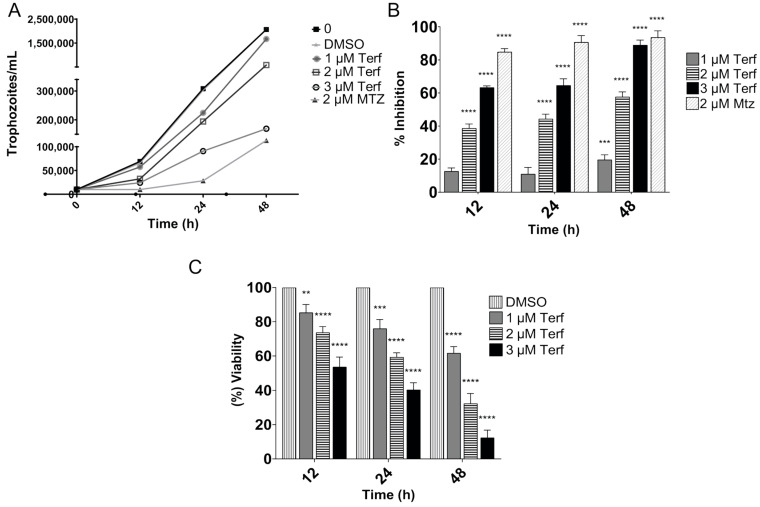
In vitro effect of terfenadine on *Giardia lamblia* trophozoites after incubation for 12, 24, and 48 h. (**A**) Growth kinetics, (**B**) percentage of growth inhibition, and (**C**) cell viability compared to DMSO. Data correspond to mean values ± SD of three independent experiments. ** *p* < 0.01, *** *p* < 0.001, **** *p* < 0.0001.

To determine the effect of terfenadine on parasite growth, we tested terfenadine at final concentrations of 1, 2, and 3 μM. The parasites without treatment showed stable growth kinetics, and parasite numbers tended to decrease in the first 12 h after treatment with all tested concentrations of terfenadine (Figure 2A). The maximum inhibitory effects were observed with 2 and 3 μM after 48 h of incubation, resulting in 57% and 88% inhibition, respectively. Terfenadine at 3 μM exhibited a similar inhibitory effect as that of metronidazole (MTZ) 2 μM (89% and 92%, respectively), with an IC_50_ = 1.6 μM (Figure 2B and Table 1). Cells without treatment and those treated with dimethyl sulfoxide (DMSO) did not show any significant differences.

Viable trophozoites at the end of the terfenadine exposition periods were determined by trypan blue exclusion. As shown in Figure 2C, after 12 h of terfenadine treatment (with 1, 2, and 3 μM, respectively), only 85%, 73%, and 53% of remanent trophozoites were viable in comparison to DMSO controls. The most dramatic effect was observed with 2 and 3 μM at 48 h; only 32% and 12%, respectively, of remanent trophozoites were viable.

### 2.2. Terfenadine Did Not Affect Caco-2 Cells but Was Selective for Giardia

**Figure 3:**
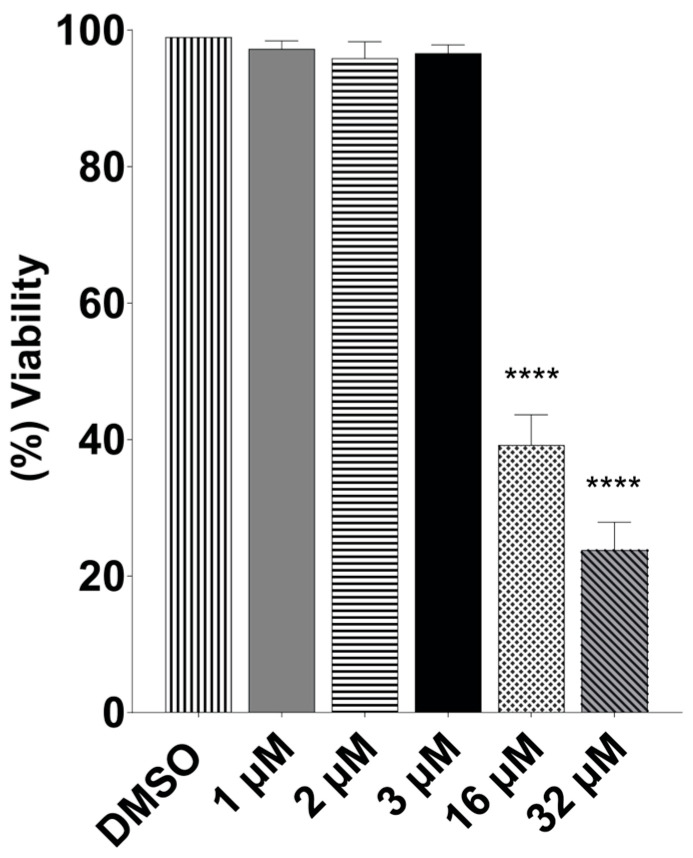
Dose–response curve for Caco-2 cell viability in the presence of terfenadine. Data correspond to mean values ± SD of three independent experiments. **** *p* < 0.0001.

To determine the specificity of terfenadine on *Giardia lamblia*, the effect of terfenadine on Caco-2 cells was evaluated. Cytotoxicity assays were performed by treating Caco-2 cells with DMSO (negative control) and 1, 2, 3, 16, and 32 μM of terfenadine for 48 h and were evaluated by MTT assay. The results showed that terfenadine at 1, 2, and 3 μM had no effect on Caco-2 cells. However, at higher concentrations tested, namely, 16 and 32 μM, an evident cytotoxic effect was observed, with viability values of 38% and 23%, respectively. In addition, a CC_50_ 16 μM and selectivity index (SI) = 10.7 were obtained (Figure 3 and Table 1).

### 2.3. Terfenadine Inhibited *Giardia lamblia* Adhesion to Caco-2 Cells

**Figure 4:**
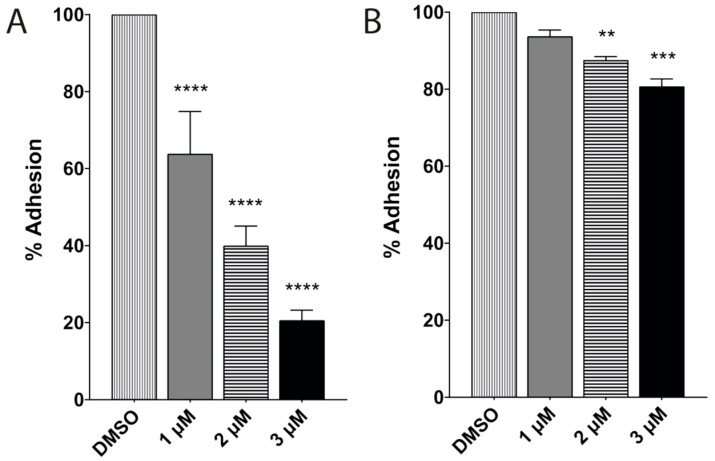
Percentage of adhesion of *Giardia lamblia* trophozoites to Caco-2 cells. Trophozoites previously exposed for 48 h to terfenadine compared to DMSO (**A**). Under standard assay conditions, in the presence of terfenadine from 2 h compared to DMSO control (**B**). Data correspond to mean values ± SD of three independent experiments. ** *p* < 0.01, *** *p* < 0.001, **** *p* < 0.0001.

When the inhibition and viability assays were performed, the parasite’s adhesion to glass was compromised. An interaction assay demonstrated that trophozoites treated with 1, 2, and 3 μM of terfenadine for 48 h, respectively, showed a 41%, 60%, and 79% reduction in adherence to Caco-2 cells (Figure 4A). The same effect was observed when the interaction assay was performed with healthy trophozoites in the presence of terfenadine. With only 2 h of drug exposure, adherence was reduced by 6.8%, 13.4%, and 18.5% with 1, 2, and 3 μM, respectively, in comparison to the DMSO control (Figure 4B).

### 2.4. Terfenadine Altered the Morphology of *Giardia lamblia* Trophozoites

**Figure 5:**
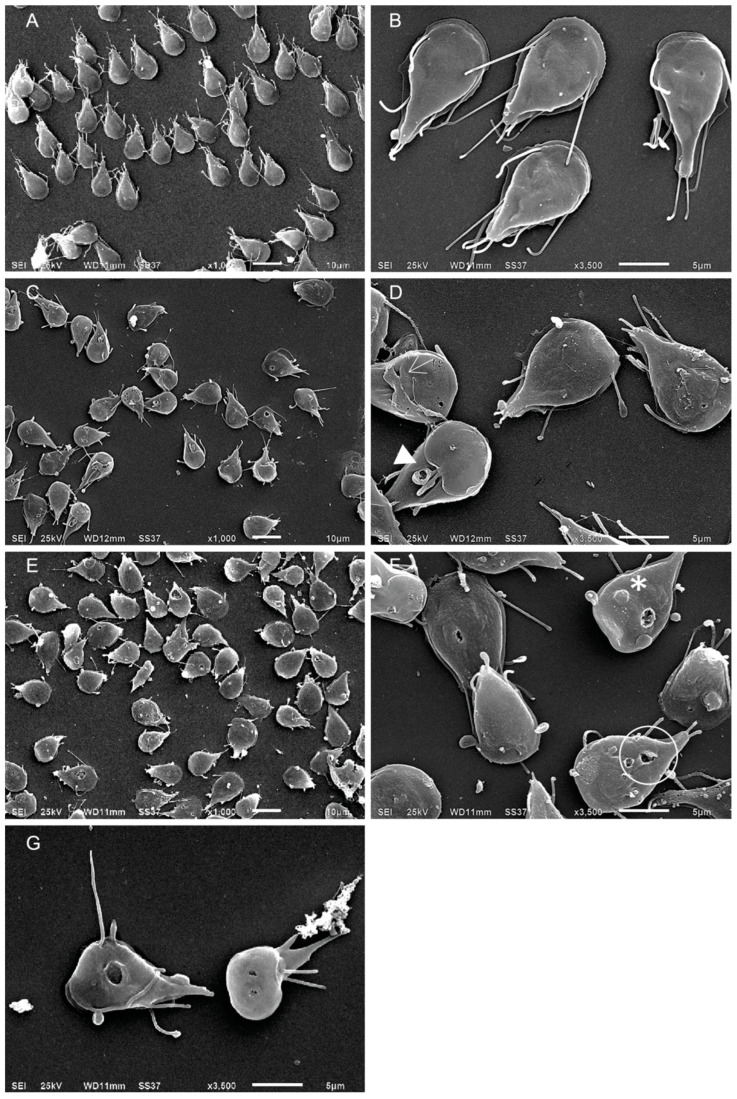
Scanning electron microscopy (SEM) micrographs of *G. lamblia* trophozoites after 48 h treatment with terfenadine. DMSO (**A**,**B**), 1 μM (**C**,**D**), 2 μM (**E**,**F**), and 3 μM (**G**). Arrowhead = damage and short flagella, asterisk = dorsal protrusions, and circle = membrane perforations. Scale bar 5 and 10 µm.

Ultrastructural changes in trophozoites after 48 h of terfenadine treatment were evaluated using scanning electron microscopy (SEM microscopy). Trophozoites treated with DMSO (negative control) presented the characteristic morphology: pyriform, ventral disc, median body, caudal zone, and flagella without any damage (Figure 5A,B). Images of trophozoites treated with terfenadine showed parasites with damage in the caudal region and loss of their pyriform shape. At 1 μM concentration, 80% of the parasite population showed damage in the ventral disc, an apparent shortening of flagella, and membrane damage (Figure 5C,D). With 2 μM, damage in the caudal region prevailed, including folding of the cell, giving the appearance of size reduction; membrane damage; and protrusions on the dorsal side of the cell. In some trophozoites, instead of a typical flagellum, an apparent extension of the cell membrane was visible, and 100% of the cells were damaged (Figure 5E,F). At 3 μM, a large amount of cellular debris was observed, and in the remaining trophozoites, structural damage was more evident, highlighting perforations in the membrane and ventral disc (Figure 5G).

### 2.5. Morphological Changes by Terfenadine Involved Remodeling of Tubulin on Giardia lamblia Trophozoites

**Figure 6:**
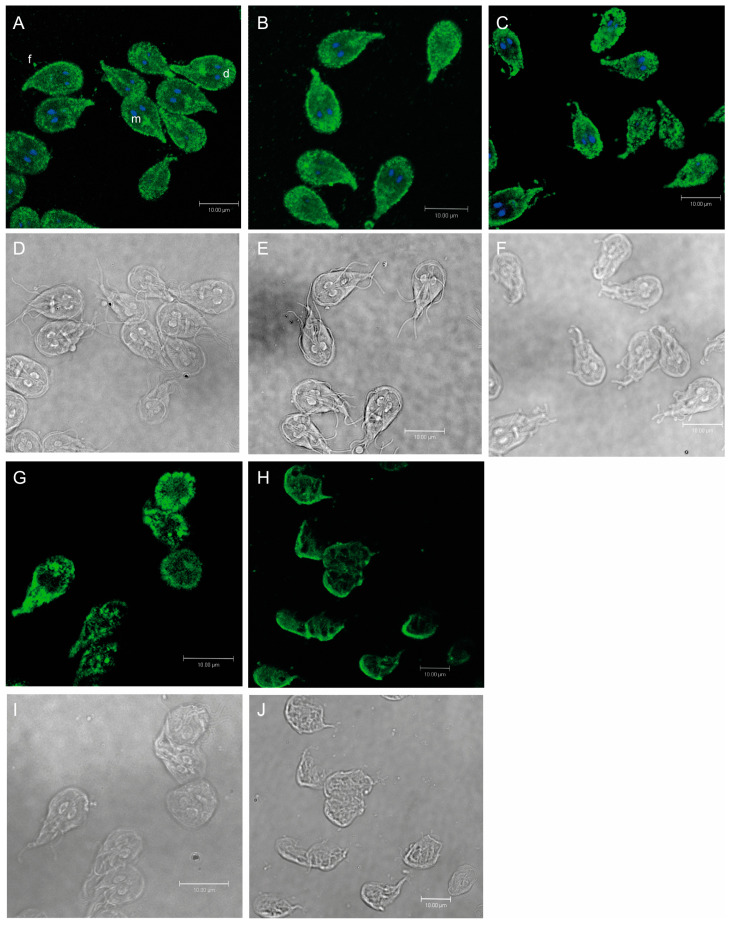
Confocal micrographs of *Giardia lamblia* trophozoites after 48 h of treatment with terfenadine. Untreated cells (**A**,**B**), DMSO (**C**,**D**), 1 μM (**E**,**F**), 2 μM (**G**,**I**), 3 μM (**H**,**J**), flagella (f), middle body (m), and (d) nuclei. Bar = 10 µm.

On the basis of the morphological damage in trophozoites due to terfenadine, we first analyzed the distribution of tubulin, the basic constituent of the *Giardia* microtubular cytoskeleton. Confocal microscopy showed tubulin cytoplasmic localization, highlighting the ventral disc, flagella, and median body, in untreated and DMSO-treated trophozoites (Figure 6A,B). Parasites treated with 1 μM terfenadine began to present changes of tubulin localization and distribution. In addition, small protein aggregates, distributed randomly throughout the cell, that are associated with morphological changes were observed (Figure 6C,F). At a concentration of 2 μM, a higher number of aggregates were present, and they had a nonrandom localization; they occupied mostly the periphery and caudal region, with an apparent decrease in cytoplasmic tubulin staining (Figure 6G). At 3 μM, where there was a greater amount of destroyed cells, the remaining cells presented reduced and nonuniform tubulin staining (Figure 6H,J).

### 2.6. Terfenadine Caused Downreglation of Tubulin in *Giardia lamblia* Trophozoites

**Figure 7:**
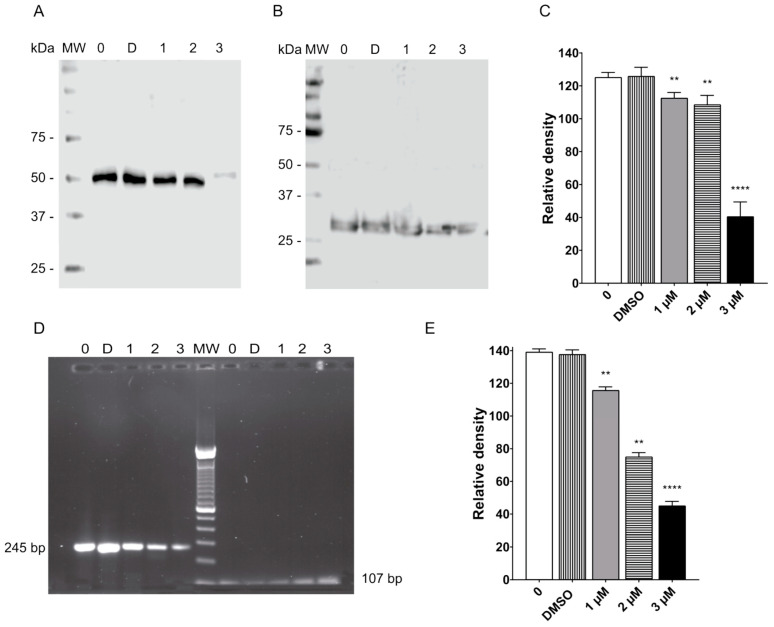
Terfenadine caused decreased protein and mRNA levels in *tubulin*. The amount of tubulin was analyzed by Western blotting (**A**). Taglin was used as a loading control (**B**). Semiquantitative densitometric analysis of Western blot (**C**). The expression of *tubulin* (254 bp) was analyzed by RT-PCR, and *shippo-1* (107 bp) was used as an internal control (**D**). Semiquantitative densitometric analysis (**E**). ** *p* < 0.01, **** *p* < 0.0001.

Because terfenadine caused tubulin reorganization, we explored whether there were changes in tubulin expression. Western blot analysis revealed that the amount of tubulin was decreased with all tested concentrations as compared to untreated and DMSO-treated cells. The major reduction in tubulin was observed in cells treated with 3 µM of terfenadine (Figure 7A). After densitometric analysis, the downregulations observed were 11%, 13%, and 68% with terfenadine at 1, 2, and 3 μM, respectively (Figure 7C).

To further support the above findings, RT-PCR was performed, and the results showed that α-tubulin was reduced significantly by 17%, 47%, and 67% after 48 h of terfenadine treatment at concentrations of 1, 2, and 3 μM, respectively, as compared to the DMSO control (Figure 7E).

### 2.7. Terfenadine Induced Alterations in GiK Expression

**Figure 8:**
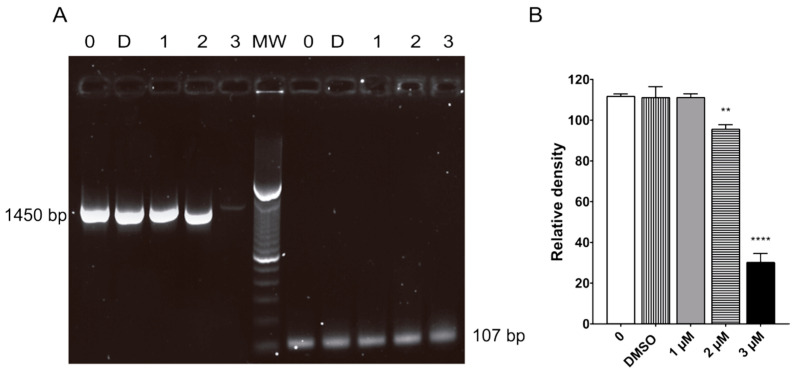
Terfenadine caused decreased mRNA levels of *gik*. RT-PCR analysis of the gene coding for GiK (1450 bp), and *shippo-1* (107 bp) was used as an internal control (**A**). Semiquantitative densitometric analysis (**B**).** *p* < 0.01, **** *p* < 0.0001.

Molecular modeling studies suggest that *GiK* is a possible target of terfenadine in *Giardia lamblia*. Here, the expression of *GiK* after terfenadine treatment was analyzed by RT-PCR. RT-PCR analysis revealed that the expression of GiK was reduced by 2%, 18%, and 74% with 1, 2, and 3 μM of terfenadine, respectively, as compared to untreated and DMSO controls (Figure 8B).

## 3. Discussion

The protozoan *Giardia lamblia*, responsible for diarrheagenic disease in animals and humans, is related to increasing rates of drug resistance and treatment failures for the most used drugs, including metronidazole and albendazole [21]. Therefore, the need for new therapies is more urgent. Currently, drug repositioning is a research approach focused on identifying new uses or therapeutic applications for existing drugs instead of spending 10–15 years developing a new drug that may not be effective or that has an investment cost that may make it unaffordable to the population. Currently, some drugs, such as disulfiram, auranofin, and acetylsalicylic acid, among others, which were initially designed for different purposes, showed promising results in *Giardia lamblia*, which need to be confirmed in people [15,22,23]. Others yielded encouraging results in human studies, e.g., auranofin, but the mode of action of this drug is still not completely understood [12]. Previously, our group described that terfenadine, a selective histamine H1-receptor antagonist, theoretically interacts principally with hydrophobic and aromatic residues at a specific site of a putative potassium channel of *Giardia lamblia* (*GiK*) [24].

In this study, on the basis of efficacy, terfenadine was as active as MTZ with an IC_50_ of 1.6 μM and SI of 10.7 (Table 1). On the basis of SI, the ideal drug should have a relatively high toxic concentration and an antigiardial activity at very low concentrations. Our SI value was 10. Therefore, we can assume that terfenadine is a selected potential drug that can be further investigated. Currently, terfenadine is no longer available on the market in several countries because of its adverse effect of prolonging the electrocardiogram QT interval [25]. However, this adverse effect is related to extended use, high doses, and concurrent antifungal drug use with ketoconazole, which inhibits CYP3A4. As a result, terfenadine does not undergo hepatic metabolism [26]. The median lethal dose (LD_50_) of terfenadine is reported to be 5000 mg/kg in mice, which is significantly higher than the doses used in our study. In addition, dogs tolerated an oral single dose of 30 mg/kg daily for up to 2 years without any side effects. This suggests that the concentrations of terfenadine used in our study are within a safe and effective range for assessing its potential as a therapeutic agent against the parasite [27]. Furthermore, in pediatric subjects with allergic rhinitis, no serious adverse effects were reported at doses of 40 mg. These findings provide crucial insights into the drug’s safety profile when used in children [28]. Additionally, a systematic literature review included a study on terfenadine’s safety for nursing mothers. The relative infant dose for terfenadine was found to be low (0.3%), indicating minimal transfer into breast milk. This suggests that terfenadine exposure through breastfeeding is low, a vital consideration for the safety of nursing infants [29].

Regarding the effect of terfenadine on *Giardia*, we observed a time–dose-dependent growth inhibition from short incubation periods (12 h). Studies with other parasites such as *Plasmodium yoelii* [30] and *Entamoeba histolytica* [18] support the antiparasitic activity of terfenadine. In *E. histolytica*, terfenadine concentrations of 1, 2, 3, and 4 μM affected not only growth but also cell viability and phagocytosis, a fundamental process for the development and survival of this parasite. These results are in part very similar to ours.

Conversely, we evaluated the cell viability (membrane integrity) of the trophozoites that survived the treatment using the exclusion staining method [31]. An interesting finding was that parasites stained blue with an apparent normal morphology, indicating that their membrane was not intact. This was corroborated by scanning microscopy. The images showed terfenadine-exposed parasites with protrusions/blisters and perforations in the membrane. Another interesting observation was that flagella could not be observed emerging from the trophozoite body. Instead, apparent membrane projections were observed, and this dramatic change in flagella has not been previously described. Taken together, these findings justify the lack of adhesion capacity of the trophozoites to the Caco-2 cells (the main mechanism of pathogenicity of *Giardia*) [32], demonstrating that terfenadine not only reduces the growth and viability of the parasites but also affects their mechanism of pathogenicity.

Several studies showed the participation of peripheral vesicles in the digestion of internalized material by *Giardia*. For example, Benchimol and coworkers (2022) described *Giardia* shape changes and the presence of large vesicles when it ingested macromolecules via receptor-mediated endocytosis, and they mentioned that these large vesicles might represent a new organelle [33]. Recently, Roberta Veríssimo and collaborators (2022) showed the presence of large protrusions in the membrane due to the effect of 4-((10H-phenothiazine-10-yl)methyl)ppaa-N-hydroxybenzamide using TEM, and they demonstrated that these protrusions corresponded to large vacuoles that harbored lamellar bodies, glycogen granules, ribosomes, and some internalized flagella, indicating that the cell may be undergoing programmed cell death similar to autophagy [34]. Conversely, a previous work with lactoferrin by Hugo Aguilar-Diaz and collaborators (2017) showed the presence of blisters and perforations in the plasma membrane of *Giardia* with unusual aggregates, suggesting that *Giardia* could be undergoing programmed cell death damage [35]. In our results, the trophozoites showed large vesicles and perforations on the plasma membrane because of terfenadine treatment.

**Figure A1:**
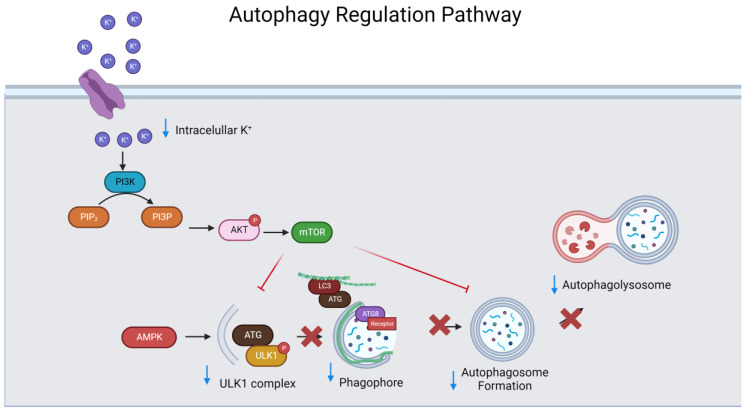
Mammalian cell’s autophagy regulation pathway.

In mammalian cells, it was described that under physiological conditions, potassium channels in the cell membrane are active, allowing the efflux of potassium ions (K^+^) from the intracellular environment to the extracellular space. This activity helps maintain an equilibrium of electric charges and membrane potential within the cell. In response to cellular stress or ionic imbalance, such as nutrient deprivation or toxin exposure, the intracellular concentration of K^+^ may change. Elevated intracellular K^+^ levels can trigger the activation of an autophagic signaling pathway. This signaling involves the regulation of key proteins, including the inhibition of the mTOR (mammalian target of rapamycin) pathway and activation of the ULK1 (unc-51-like kinase 1) complex. Increased intracellular potassium levels can lead to the inhibition of the mTOR protein, which typically suppresses autophagy. Inhibition of mTOR alleviates its inhibitory effect on ULK1. Activation of ULK1 is critical for the initiation of phagophore formation, the membranous structure that engulfs cellular cargos targeted for autophagic degradation. With active ULK1, phagophore formation begins. This process involves the conjugation of autophagic proteins, such as LC3 (microtubule-associated protein 1 light chain 3) and Atg12, to the phagophore membrane. The degradation of cellular cargos within the autophagolysosome releases nutrients and can lead to programmed cell death (Appendix B, Figure A1) [36,37,38,39].

In *Giardia*, bioinformatic analysis revealed the presence of genes associated with autophagy such as TOR, S6K1, PI3K, Atg1, Atg16, Atg7, Atg8, and Atg18. The overall mechanism of autophagy begins with the formation of a phagophore. Once the autophagosome is ready, it is transferred toward the lysosome across microtubules [36,37,40]. The integrity of the tubulin cytoskeleton is necessary to control exocytosis/endocytosis events. As a result, excessive autophagy occurs in *Giardia*, in which the activity of ATG proteins, a group of proteins involved in phagosome formation such as LC3 (microtubule-associated protein 1A/1B light chain 3) and ATG8, is increased. These proteins coordinate the formation of the autophagosome membrane with microtubule recruitment and subsequent degradation after fusion with the lysosome [38,41,42,43].

Previously, in human cells, it was elucidated that the interaction of HERG voltage-dependent potassium channels with PiP2 helps in regulating the activity of these channels [44,45]. Currently, there is no direct evidence that shows a direct relationship between ion channels and tubulin in *Giardia*. However, studies such as those of Melgari, Camacho, and Vitre [46,47,48] reported a whole signaling cascade by which the dynamics of the cytoskeleton of these human cells can be modified by the alteration of an ion channel.

**Figure A2:**
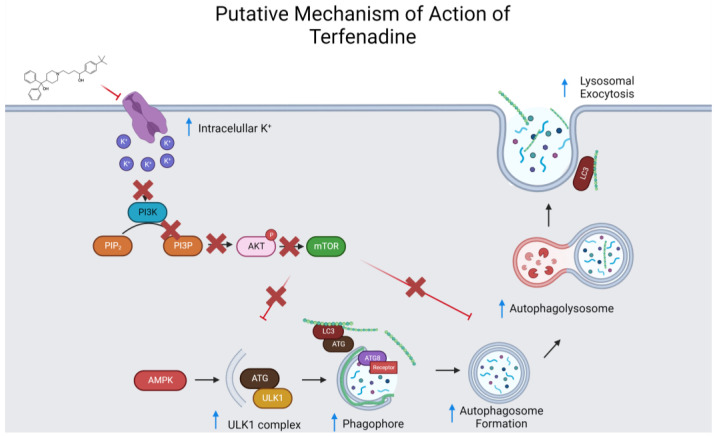
Putative mechanism of action of terfenadine: induction of autophagy. Terfenadine is thought to induce autophagy by inhibiting cell membrane potassium channels, causing an intracellular potassium buildup and ionic stress. This stress, along with other cellular signals, inhibits the mTOR pathway, which typically suppresses autophagy. In response, the ULK1 complex is activated, initiating phagophore formation, the initial step in autophagosome creation. During this process, autophagic proteins like LC3 (microtubule-associated protein 1 light chain 3) and Atg12 are added to the phagophore membrane. Completed phagophores merge with lysosomes, forming autophagolysosomes where cellular contents are degraded and recycled, ultimately inducing autophagy. This represents a putative mechanism by which terfenadine triggers autophagy by affecting potassium channels and initiating a series of intracellular events.

In this work, we observed tubulin restructuring and the deregulation of protein expression by terfenadine treatment, concomitant with the deregulation of *GiK*. Our results suggest *Giardia* programmed cell death by autophagy (Appendix C, Figure A2).

## 4. Materials and Methods

### 4.1. Cell Culture of Giardia lamblia Trophozoites and Caco-2 Cell Line

Trophozoites of *Giardia lamblia* (WB clone C6) were grown axenically at 37 °C in borosilicate culture tubes containing Diamond’s TYI-S-33 modified medium (Appendix A, Table A1) at pH 7.0 (supplemented with 0.5 mg/mL of bovine bile and 10% fetal bovine serum) [49]. Cultures were maintained by cell subculturing at intervals of 72 or 96 h. Briefly, trophozoites were detached by incubation in an ice-water bath for about 20 min, then 0.5–1.0 µL of the cell suspension was transferred to culture tubes filled with fresh medium and incubated at 37 °C. Human colon carcinoma cells from (Caco-2) were cultured at 37 °C in Dulbecco’s modified Eagle’s culture medium (DMEM) supplemented with 10% fetal bovine serum (FBS; Invitrogen) in a humidified atmosphere (5% CO_2_ and 95% air). For routine maintenance, cells were split twice a week by detachment with 0.25% trypsin–0.025% EDTA and reseeded in 25 cm^2^ flasks in a split ratio of 1:4. For experiments, the numbers of Caco-2 cells and trophozoites were estimated by counting in a Neubauer chamber [50].

### 4.2. Growth Inhibition Assay

To evaluate the effect of terfenadine on *Giardia lamblia* growth, 10,000 parasites/mL were grown in TYI-S-33 medium containing 1, 2, and 3 μM of terfenadine (Sigma-Aldrich St. Louis, MO, USA). Cultures were monitored at 12, 24, and 48 h. Untreated cells and dimethyl sulfoxide (0.09% DMSO, Sigma-Aldrich, Saint Louis, MO, USA), a drug diluent, were used as negative controls. Metronidazole (2 µM MTZ) was used as a positive control. After the incubation periods, cell culture tubes were cooled in an ice-water bath to release adhered trophozoites. Cell density was calculated using a Neubauer chamber. Percentages of inhibition were calculated in comparison with DMSO control.

### 4.3. Terfenadine Cytotoxicity Assay

The cytotoxicity of terfenadine was evaluated on the Caco-2 cell line by 3-(4,5-dimethylthiazol-2-yl)-2,5-diphenyltetrazolium bromide (MTT) assay, based on a reduction in MTT to purple formazan granules. The MTT assay was performed as described previously by Mossman [51], with slight adjustments. Briefly, Caco-2 cells were seeded into a 96-well plate (5000 cells/well) and grown at 37 °C in 5% CO_2_ for 24 h. Cells were then treated with DMSO (0.09%, the negative control) or terfenadine (1, 2, 3, 16, and 32 μM). After 24 h, the medium was removed, 100 µL of MTT reagent (0.8 mg/mL in serum-free medium) was added to each well, and the cells were incubated at 37 °C for 4 h in 5% CO_2_–95% air atmosphere. Next, the medium was replaced with DMSO (150 µL) to dissolve the formazan crystals, and the absorbance was measured at 570 nm on a microplate reader (Multiskan SkyHigh Microplate Spectrophotometer, A51119500C, Thermo Scientific, Waltham, MA, USA).

### 4.4. Selectivity Index Calculation

The calculation of 50% cytotoxicity for both Caco-2 cells (CC_50_ Caco-2) and *Giardia lamblia* (IC_50_ *Giardia*) was conducted using nonlinear regression. The selective index (SI) was calculated as follows [52]:

SI = CC_50_ Caco-2/IC_50_ *Giardia lamblia*

### 4.5. Cell Viability Assay

A dye exclusion test was used to determine the viability of trophozoites after DMSO or terfenadine treatment. Approximately 10 µL of the culture was mixed with 10 µL of trypan blue (0.4% Gibco-BRL). The number of viable and/or dead cells was determined by light microscopy and then calculated as a percentage of negative control cells.

### 4.6. Cell Adhesion Assay

To evaluate the possible effect of terfenadine on adhesion, *Giardia* trophozoites were incubated with Caco-2 intestinal cells [53]. Briefly, 1.0 × 10^4^ cells/well were cultured in a 24-well microplate and maintained in a humidified atmosphere of 5% CO_2_ at 37 °C until cells reached the monolayer. The experimental conditions were as follows: (1) interaction of trophozoites previously treated with DMSO (0.09%, negative control) and terfenadine (1, 2, 3 μM) for 48 h, with Caco-2 cells, and (2) interaction of trophozoites with Caco-2 cells and terfenadine at the same time. The trophozoites were incubated with monolayers at a Caco-2 cells: *Giardia lamblia* trophozoites ratio of 4:1 in 1 mL of TYI-S-33 medium. After 2 h of cell interaction at 37 °C, the medium was removed, the plates were kept on ice, and wells were filled with cold phosphate-buffered saline (PBS) and placed on ice for 30 min to detach the adherent trophozoites. The numbers of adherent and nonadherent trophozoites was determined by counting in a Neubauer chamber. The effect on adherence was expressed as the percentage of adhered trophozoites in relation to the total number of cells, and the results obtained were compared with control cultures.

### 4.7. Scanning Electron Microscopy (SEM)

To analyze the morphology of *Giardia lamblia*, 10,000 parasites/mL were grown in TYI-S-33 medium containing 1, 2, or 3 μM of terfenadine or 0.09% DMSO (negative control) for 48 h. After treatment, parasites were harvested after 48 h by centrifugation (10 min at 1973× *g* at 4 °C), then washed twice with PBS, fixed for 1 h with 2.5% glutaraldehyde (Sigma-Aldrich, St. Louis, MO, USA) in PBS, and adhered to poly-L-lysine-coated coverslips (Sigma-Aldrich, St. Louis, MO, USA). Coverslips with parasites adhered were washed three times with PBS and postfixed with 1% OsO_4_ in PBS for 1 h. Next, parasites were washed three times with PBS, dehydrated in gradient of ethanol series (50–100%), and subjected to critical point drying with CO_2_ in a Samdry-780 dryer (Tousimis Research, Rockville, MD, USA). Finally, cells were mounted on stainless steel holders and sputter-coated with a thin layer of gold in a Denton Vacuum Desk II (Denton Vacuum, Moorestown, NY, USA). Samples were examined and photographed using an SEM JSM-6510-LV (JEOL Ltd., Tokyo, Japan).

### 4.8. Immunofluorescence

Parasites treated with DMSO (the negative control) or terfenadine (1, 2, 3 μM) were harvested after 48 h by centrifugation (10 min at 1973× *g* at 4 °C), washed twice in PBS, and allowed to attach to polyethylenimine-coated coverslips for 20 min. The coverslips were fixed with methanol–acetone (Sigma-Aldrich, St. Louis, MO, USA) in a 1:1 ratio at −20 °C for 10 min. The adhered cells were also permeabilized with 0.05% Triton X-100 (in PBS) for 30 min. After two washes with PBS, cells were incubated for 1 h with 1% bovine serum albumin (BSA) to block nonspecific binding. Next, cells were incubated with diluted 1:1000 mouse anti-α-tubulin antibody (Invitrogen, Thermo Fisher, Scientific, Waltham, MA, USA) for 1 h at room temperature. After two PBS washes, the cells were incubated with goat anti-mouse IgG and FITC conjugated (1:200 dilution, Thermo Fisher Scientific). The coverslips were washed 10 times in PBS and then mounted on microscope slides with a drop of mounting medium containing DAPI (Prolong Gold Invitrogen). The cells were analyzed using a Leica confocal microscope (Leica TCS SP8, Confocal Laser Scanning Microscope), and images were processed using Leica Lite Software.

### 4.9. SDS-PAGE and Western Blot Assay

#### 4.9.1. Protein Extracts and SDS-PAGE

Cells treated with DMSO (the negative control) or terfenadine (1, 2, 3 μM) cells were harvested after 48 h and processed to obtain a total protein extract according to an earlier report [54]. Briefly, the parasites were collected by cooling and posterior centrifugation at 1973× *g* for 10 min at 4 °C. The cells were lysed with RIPA buffer (sodium chloride, 150 mM, Tris-HCL, 50 mM, Nonidet P-40 1%, sodium deoxycholate 0.5%, SDS 0.1%) supplemented with complete protease inhibitor (Roche), PMSF (Sigma-Aldrich St. Louis, MO, USA), and sodium orthovanadate (Sigma-Aldrich St. Louis, MO, USA) and were incubated on ice for 30 min. Protein concentration was determined using a Bradford assay (Pierce Detergent Compatible Bradford Assay, Thermo Fisher Scientific). Readings were made using a microplate reader at 595 nm (Multiskan SkyHigh Microplate Spectrophotometer, A51119500C, Thermo Scientific, Waltham, MA, USA). Protein extracts (25 µg) were separated under reducing conditions by electrophoresis (polyacrylamide gel electrophoresis in the presence of sodium dodecyl sulfate, SDS-PAGE) (10%) [55]. The electrophoretic separation of the proteins was carried out at a constant voltage of 120 V for 2 h. Visualization of protein bands was carried out by incubating the gel with a Coomassie staining solution.

#### 4.9.2. Western Blot

Following SDS-PAGE, proteins were transferred to PVDF membranes (Amersham Pharmacia Biotech, Little Chalfont, UK) at 300 A for 70 min with a Transblot apparatus (Bio-Rad, Hercules, CA, USA). After transfer, the membranes were blocked using Pierce Fast Blocking buffer solution 1X (Thermo Fisher Scientific) for 20 min. After three washes with PBS containing 0.05% Tween 20 (PBS-T), the membranes were incubated with 1:500 mouse anti-α-tubulin (Invitrogen, #13-8000, Carlsbad, CA, USA) for 2 h. Membranes were washed three times and incubated for 1 h with goat anti-mouse IgG antibody coupled to horseradish peroxidase (1:20,000) (Pierce, Waltham, MA, USA). After five 15 min washes with PBS-T, the signal was detected by chemiluminescence (ECL Immobilon Western, Millipore, Burlington, MA, USA). Taglin 1:500 was used as a protein-loading control [56]. Signals were detected using the C-Digits system. Semiquantitative determination was performed with Image Studio Digits Software version 5.2.

### 4.10. RT-PCR

Total RNA was obtained from trophozoites treated with DMSO (the negative control) or terfenadine (1, 2, 3 μM) using a Total RNA Purification kit (NORGEN), following the manufacturer’s instructions. cDNAs were obtained by a reverse transcriptase reaction (Maxima cDNA Synthesis Kit, Thermo Fisher Scientific) using 1 μg of RNA and Oligo dt_20_ primer (Integrated DNA). For RT-PCR, the primers were designed using the following GenBank *Giardia* sequences: *GiK* (GenBank accession no. XM_001709438.1) and *α-tubulin* (GenBank accession no. XM_001705668.1). *GiK* and *α-Tubulin* primer sequences were as follows: *GiK*-F 5′-GCA CTG CAG CAG GTT AAG CTA TC-3′, *GiK*-R 5′-GAG TCT AGA AAA TTG TTT AAA TAA ATC AAC GC-3′ *tubulin*-F 5′-GCA GCTGAT CTC TGG CAA GGA-3′, and *tubulin*-R 5′-GCG AGG GGT AGACGA CGA ACT C-3′. The expression of the abovementioned genes was normalized to the expression level of *shippo 1* (GenBank accession no. XM_001708537.1) using the following primers: *shippo 1*-F 5′-CGT CAT CAA CAG GTC CGA-3′ and *shippo 1*-R 5′-CCA GCT CTC CTT GAA CAC-3′. The RT-PCR conditions included an initial denaturation at 95 °C for 2 min and 35 cycles of 95 °C for 35 s; 56 °C (*tubulin*), 59 °C (*GiK*), or 55 °C (*shippo 1*) for 30 s; and 72 °C for 1 min. A final extension of 7 min at 72 °C was performed.

### 4.11. Statistical Analysis

All experiments were performed in triplicate and repeated three different times. Data were analyzed by ANOVA and post hoc Dunnett’s multiple comparisons test. *p* values of ≤0.05 were considered statistically significant (GraphPad Prism version 9.0 for Windows, Software, La Jolla, CA, USA, was employed). Error bars in graphics indicate standard deviations for the experiments.

## 5. Conclusions

Our data support the hypothesis that terfenadine could be a promising selective candidate for the treatment of giardiasis. Further studies are necessary to establish autophagy as a possible mechanism of action of terfenadine in *Giardia lamblia*.

## Figures and Tables

**Table 1:** Antigiardial cytotoxicity and selectivity index values of terfenadine.

IC_50_ on *Giardia*	CC_50_ on Caco-2 Cells	Selectivity Index CC_50_ on Caco-2 Cells/IC_50_ on *Giardia*
1.6 μM	16 μM	10.7

## Data Availability

Data is contained within the article.

## Appendix A

**Table A1:** TYI-S-33 modified medium.

D-glucose	DIBICO 2001-E
NaCl	Sigma-Aldrich S7653
K2HPO4	Sigma-Aldrich P5655
KH2PO4	Sigma-Aldrich P5505
Peptone	Sigma-Aldrich G8270
L-ascorbic acid	Sigma-Aldrich A5960
L-cysteine	Sigma-Aldrich C6852
Ferric ammonium citrate	Sigma-Aldrich F5879
Dehydrated bovine bile	Sigma-Aldrich B3883

## References

[1] Lalle M., Hanevik K. (2018). Treatment-refractory giardiasis: Challenges and solutions. Infect. Drug Resist..

[2] Leung A.K.C., Leung A.A.M., Wong A.H.C., Sergi C.M., Kam J.K.M. (2019). Giardiasis: An Overview. Recent Pat. Inflamm. Allergy Drug Discov..

[3] Halliez M.C., Buret A.G. (2013). Extra-intestinal and long term consequences of *Giardia duodenalis* infections. World J. Gastroenterol..

[4] Painter J.E., Collier S.A., Gargano J.W. (2017). Association between *Giardia* and arthritis or joint pain in a large health insurance cohort: Could it be reactive arthritis?. Epidemiol. Infect..

[5] Watkins R.R., Eckmann L. (2014). Treatment of giardiasis: Current status and future directions. Curr. Infect. Dis. Rep..

[6] Vivancos V., González-Alvarez I., Bermejo M., Gonzalez-Alvarez M. (2018). Giardiasis: Characteristics, Pathogenesis and New Insights About Treatment. Curr. Top. Med. Chem..

[7] Hernández Ceruelos A., Romero-Quezada L.C., Ruvalcaba Ledezma J.C., López Contreras L. (2019). Therapeutic uses of metronidazole and its side effects: An update. Eur. Rev. Med. Pharmacol. Sci..

[8] Piloiu C., Dumitrascu D.L. (2021). Albendazole-Induced Liver Injury. Am. J. Ther..

[9] (2020). Tinidazole. LiverTox: Clinical and Research Information on Drug-Induced Liver Injury.

[10] Chen Y., Li P., Chen T., Liu H., Wang P., Dai X., Zou Q. (2023). Ronidazole Is a Superior Prodrug to Metronidazole for Nitroreductase-Mediated Hepatocytes Ablation in Zebrafish Larvae. Zebrafish.

[11] Ansell B.R., McConville M.J., Ma’ayeh S.Y., Dagley M.J., Gasser R.B., Svärd S.G., Jex A.R. (2015). Drug resistance in *Giardia duodenalis*. Biotechnol. Adv..

[12] Argüello-García R., Leitsch D., Skinner-Adams T., Ortega-Pierres M.G. (2020). Drug resistance in *Giardia*: Mechanisms and alternative treatments for Giardiasis. Adv. Parasitol..

[13] Leitsch D. (2015). Drug Resistance in the Microaerophilic Parasite *Giardia lamblia*. Curr. Trop. Med. Rep..

[14] Castillo-Villanueva A., Rufino-Gonzalez Y., Mendez S.T., Torres-Arroyo A., Ponce-Macotela M., Martinez-Gordillo M.N., Reyes-Vivas H., Oria-Hernandez J. (2017). Disulfiram as a novel inactivator of *Giardia lamblia* triosephosphate isomerase with antigiardial potential. Int. J. Parasitol. Drugs Drug Resist..

[15] Ochoa-Maganda V.Y., Rangel-Castañeda I.A., Suárez-Rico D.O., Cortés-Zárate R., Hernández-Hernández J.M., Pérez-Rangel A., Chiquete-Félix N., León-Ávila G., González-Pozos S., Gaona-Bernal J. (2020). Antigiardial Activity of Acetylsalicylic Acid Is Associated with Overexpression of HSP70 and Membrane Transporters. Pharmaceuticals.

[16] Tejman-Yarden N., Miyamoto Y., Leitsch D., Santini J., Debnath A., Gut J., McKerrow J.H., Reed S.L., Eckmann L. (2013). A reprofiled drug, auranofin, is effective against metronidazole-resistant *Giardia lamblia*. Antimicrob. Agents Chemother..

[17] Mertens R.T., Gukathasan S., Arojojoye A.S., Olelewe C., Awuah S.G. (2023). Next Generation Gold Drugs and Probes: Chemistry and Biomedical Applications. Chem. Rev..

[18] Rangel-Castañeda I.A., Castillo-Romero A., León-Ávila G., Zermeño-Ruiz M., Hernández-Hernández J.M. (2021). Drug repositioning: Antiprotozoal activity of terfenadine against Entamoeba histolytica trophozoites. Parasitol. Res..

[19] Palomo-Ligas L., Gutiérrez-Gutiérrez F., Ochoa-Maganda V.Y., Cortés-Zárate R., Charles-Niño C.L., Castillo-Romero A. (2019). Identification of a novel potassium channel (GiK) as a potential drug target in *Giardia lamblia*: Computational descriptions of binding sites. PeerJ.

[20] Information NCoB (2023). Terfenadine Pubchem: NCBI. https://pubchem.ncbi.nlm.nih.gov/compound/Terfenadine.

[21] Mørch K., Hanevik K. (2020). Giardiasis treatment: An update with a focus on refractory disease. Curr. Opin. Infect. Dis..

[22] Tejman-Yarden N., Eckmann L. (2011). New approaches to the treatment of giardiasis. Curr. Opin. Infect. Dis..

[23] Ehrenkaufer G., Li P., Stebbins E.E., Kangussu-Marcolino M.M., Debnath A., White C.V., Moser M.S., DeRisi J., Gisselberg J., Yeh E. (2020). Identification of anisomycin, prodigiosin and obatoclax as compounds with broad-spectrum anti-parasitic activity. PLoS Negl. Trop. Dis..

[24] Roy M., Dumaine R., Brown A.M. (1996). HERG, a primary human ventricular target of the nonsedating antihistamine terfenadine. Circulation.

[25] Cataldi M., Maurer M., Taglialatela M., Church M.K. (2019). Cardiac safety of second-generation H(1) -antihistamines when updosed in chronic spontaneous urticaria. Clin. Exp. Allergy.

[26] Honig P.K., Wortham D.C., Zamani K., Conner D.P., Mullin J.C., Cantilena L.R. (1993). Terfenadine-ketoconazole interaction. Pharmacokinetic and electrocardiographic consequences. JAMA.

[27] Gibson J.P., Huffmann K.W., Newberne J.W. (1982). Preclinical safety studies with terfenadine. Arzneimittelforschung.

[28] Simons F.E., Watson W.T., Simons K.J. (1987). The pharmacokinetics and pharmacodynamics of terfenadine in children. J. Allergy Clin. Immunol..

[29] Ngo E., Spigset O., Lupattelli A., Panchaud A., Annaert P., Allegaert K., Nordeng H. (2022). Antihistamine use during breastfeeding with focus on breast milk transfer and safety in humans: A systematic literature review. Basic Clin. Pharmacol. Toxicol..

[30] Singh N., Puri S.K. (1998). Causal prophylactic activity of antihistaminic agents against Plasmodium yoelii nigeriensis infection in Swiss mice. Acta Trop..

[31] Strober W. (2015). Trypan Blue Exclusion Test of Cell Viability. Curr. Protoc. Immunol..

[32] Certad G., Viscogliosi E., Chabé M., Cacciò S.M. (2017). Pathogenic Mechanisms of *Cryptosporidium* and *Giardia*. Trends Parasitol..

[33] Benchimol M., Gadelha A.P., de Souza W. (2022). Unusual Cell Structures and Organelles in *Giardia intestinalis* and Trichomonas vaginalis Are Potential Drug Targets. Microorganisms.

[34] Oliveira R.V.F., de Souza W., Vögerl K., Bracher F., Benchimol M., Gadelha A.P.R. (2022). In vitro effects of the 4-[(10H-phenothiazin-10-yl)methyl]-N-hydroxybenzamide on *Giardia intestinalis* trophozoites. Acta Trop..

[35] Aguilar-Diaz H., Canizalez-Roman A., Nepomuceno-Mejia T., Gallardo-Vera F., Hornelas-Orozco Y., Nazmi K., Bolscher J.G., Carrero J.C., Leon-Sicairos C., Leon-Sicairos N. (2017). Parasiticidal effect of synthetic bovine lactoferrin peptides on the enteric parasite *Giardia intestinalis*. Biochem. Cell Biol..

[36] Choi S., Houdek X., Anderson R.A. (2018). Phosphoinositide 3-kinase pathways and autophagy require phosphatidylinositol phosphate kinases. Adv. Biol. Regul..

[37] Bagchi S., Oniku A.E., Topping K., Mamhoud Z.N., Paget T.A. (2012). Programmed cell death in *Giardia*. Parasitology.

[38] Abuammar H., Bhattacharjee A., Simon-Vecsei Z., Blastyák A., Csordás G., Páli T., Juhász G. (2021). Ion Channels and Pumps in Autophagy: A Reciprocal Relationship. Cells.

[39] Nicolau-Galmés F., Asumendi A., Alonso-Tejerina E., Pérez-Yarza G., Jangi S.M., Gardeazabal J., Arroyo-Berdugo Y., Careaga J.M., Díaz-Ramón J.L., Apraiz A. (2011). Terfenadine induces apoptosis and autophagy in melanoma cells through ROS-dependent and -independent mechanisms. Apoptosis.

[40] Yichoy M., Duarte T.T., De Chatterjee A., Mendez T.L., Aguilera K.Y., Roy D., Roychowdhury S., Aley S.B., Das S. (2011). Lipid metabolism in *Giardia*: A post-genomic perspective. Parasitology.

[41] Sarbassov D.D., Ali S.M., Kim D.H., Guertin D.A., Latek R.R., Erdjument-Bromage H., Tempst P., Sabatini D.M. (2004). Rictor, a novel binding partner of mTOR, defines a rapamycin-insensitive and raptor-independent pathway that regulates the cytoskeleton. Curr. Biol..

[42] Mackeh R., Perdiz D., Lorin S., Codogno P., Poüs C. (2013). Autophagy and microtubules—New story, old players. J. Cell Sci..

[43] Trisciuoglio D., Degrassi F. (2021). The Tubulin Code and Tubulin-Modifying Enzymes in Autophagy and Cancer. Cancers.

[44] Chen Y., Yu L. (2017). Recent progress in autophagic lysosome reformation. Traffic.

[45] Popova J.S., Greene A.K., Wang J., Rasenick M.M. (2002). Phosphatidylinositol 4,5-bisphosphate modifies tubulin participation in phospholipase Cbeta1 signaling. J. Neurosci..

[46] Melgari D., Barbier C., Dilanian G., Rücker-Martin C., Doisne N., Coulombe A., Hatem S.N., Balse E. (2020). Microtubule polymerization state and clathrin-dependent internalization regulate dynamics of cardiac potassium channel: Microtubule and clathrin control of K(V)1.5 channel. J. Mol. Cell. Cardiol..

[47] Camacho J., Sánchez A., Stühmer W., Pardo L.A. (2000). Cytoskeletal interactions determine the electrophysiological properties of human EAG potassium channels. Pflug. Arch..

[48] Vitre B., Coquelle F.M., Heichette C., Garnier C., Chrétien D., Arnal I. (2008). EB1 regulates microtubule dynamics and tubulin sheet closure in vitro. Nat. Cell Biol..

[49] Keister D.B. (1983). Axenic culture of *Giardia lamblia* in TYI-S-33 medium supplemented with bile. Trans. R. Soc. Trop. Med. Hyg..

[50] Bastidas O. (2013). Cell Counting with Neubauer Chamber, Basic Hemocytometer Usage.

[51] Mosmann T. (1983). Rapid colorimetric assay for cellular growth and survival: Application to proliferation and cytotoxicity assays. J. Immunol. Methods.

[52] Krzywik J., Mozga W., Aminpour M., Janczak J., Maj E., Wietrzyk J., Tuszyński J.A., Huczyński A. (2020). Synthesis, Antiproliferative Activity and Molecular Docking Studies of Novel Doubly Modified Colchicine Amides and Sulfonamides as Anticancer Agents. Molecules.

[53] Rigothier M.C., Coconnier M.H., Servin A.L., Gayral P. (1991). A new in vitro model of Entamoeba histolytica adhesion, using the human colon carcinoma cell line Caco-2: Scanning electron microscopic study. Infect. Immun..

[54] Palomo-Ligas L., Estrada-Camacho J., Garza-Ontiveros M., Vargas-Villanueva J.R., Gutiérrez-Gutiérrez F., Nery-Flores S.D., Cañas Montoya J.A., Ascacio-Valdés J., Campos-Muzquiz L.G., Rodriguez-Herrera R. (2022). Polyphenolic extract from Punica granatum peel causes cytoskeleton-related damage on *Giardia lamblia* trophozoites in vitro. PeerJ.

[55] Matsumoto H., Haniu H., Komori N. (2019). Determination of Protein Molecular Weights on SDS-PAGE. Methods Mol. Biol..

[56] Ward H.D., Lev B.I., Kane A.V., Keusch G.T., Pereira M.E. (1987). Identification and characterization of taglin, a mannose 6-phosphate binding, trypsin-activated lectin from *Giardia lamblia*. Biochemistry.

